# Is it the decision of women to choose a cesarean section as the mode of birth? A review of literature on the views of stakeholders

**DOI:** 10.1186/s12884-019-2440-2

**Published:** 2019-08-09

**Authors:** Alice Yuen Loke, Louise Davies, Yim-wah Mak

**Affiliations:** School of Nursing, The Hong Kong Polytechnic University, Hung Hom, Hong Kong

**Keywords:** Cesarean, Choice of birth, Maternal request, Decision-making

## Abstract

**Background:**

A debate on the decision of women to choose a cesarean section as the mode of birth in uncomplicated pregnancies from the views of relevant stakeholders.

**Main text:**

Using five electronic databases, a literature search was conducted for studies published from January 2003 to December 2016. Studies on a woman’s right to request or to choose a cesarean section as the mode of birth in uncomplicated pregnancies were included. Fifty-five articles were identified (39 research studies and 16 opinion-based articles). Among health professionals, obstetricians were the most supportive of this right. It is argued that although women reported wanting to choose the mode of birth, with the safety of their babies as the priority, they also relied on the advice of their maternity care provider and considered it the responsibility of their obstetrician to make the decision. A higher proportion of the general public in countries with well-developed private healthcare accepted that a woman should have the freedom to choose the mode of birth.

**Conclusions:**

This review provided a debate on the choice of pregnant women in uncomplicated pregnancies on the mode of birth from various stakeholders. Further research is required to explore what the meanings of autonomy of pregnant women to choose the mode of birth, and the process that they go through when making this decision.

**Electronic supplementary material:**

The online version of this article (10.1186/s12884-019-2440-2) contains supplementary material, which is available to authorized users.

## Background

The movement towards advocating patient involvement in the making of healthcare decisions has led to an increase in the number of women making decisions regarding their maternity care. Discussions over the involvement of women have evolved from issues such as antenatal screening tests and the use of analgesics in labor to the present debate over requests from pregnant women to undergo a cesarean section [1].

Until recently, women were expected to deliver their babies vaginally if their pregnancy was considered to be low risk. Obstetricians perform a cesarean section (CS) only if potential complications are identified in the antenatal period or emerge during labor. In recent years, women have been claimed to be making autonomous decisions on the mode of birth (MOB) of their babies, based on their preference or as a matter of “informed consent” [2]. The notion that women with low-risk pregnancies should have the right to make decisions on their MOB has become a controversial topic. The debate has focused on the appropriateness of the request for a CS where there are no medical indications or, as it has been dubbed, “a cesarean section per maternal request” (CSMR).

Numerous studies have been conducted exploring the perceptions of various stakeholders, including maternity care providers (obstetricians and midwives), pregnant women, and the general public, on the involvement of women in making decisions on a cesarean section as the mode of birth in uncomplicated pregnancies. However, there is no literature review that gives an overall picture of this controversial phenomenon in maternity care.

### Aims and objectives

The purpose of this literature review is to explore the decision of women with low-risk pregnancies to undergo a cesarean section as the mode of birth from the views of different stakeholders. The results will provide a clear and comprehensive picture of these views and their implications for maternity services.

### Methods of literature search

A literature search was undertaken to identify relevant studies on opinions of the women and other relevant stakeholders on women’s request to undergo a cesarean section in the antenatal period for uncomplicated pregnancies. The search included only English language, full-text articles from 2003 to December 2016 in the following electronic databases: MEDLINE, Science Direct, SCOPUS, CINAHL, and MIDIRS. The year 2003 was selected as the starting year for the search of relevant published articles because the American College of Obstetricians and Gynecologists had published a Committee Opinion statement in 2003 [3]. Key words for the literature search included “choice” OR “decision-making” OR “option” OR “preference”, AND “mode of birth” OR “mode of birth” OR “maternal request cesarean” OR “cesarean per maternal request”. An author search and hand search of the reference lists of the included literature uncovered three additional papers.

**Fig. 1:**
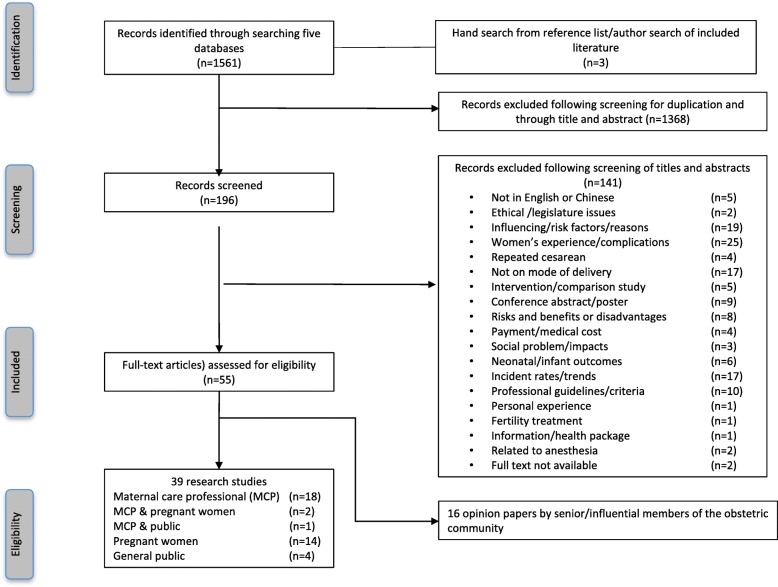
Flow chart of the study selection process

A total of 1561 articles were identified. After duplicates were removed and titles and abstracts were screened, 196 articles remained. The articles were then retrieved and read carefully to identify those eligible for inclusion in the review. Excluded were studies involving women who had previously undergone a CS, or women with medical indications for CS during the antenatal or intrapartum period. Only 39 articles met the criterion for inclusion, of focusing on views surrounding decision-making around the MOB in uncomplicated pregnancies. Opinion-based articles (*n* = 16), including commentaries and editorials, were retained to include the views of maternity care providers (MCP) and their organizations. In the end, 55 articles were included in this review. The procedures for selecting the studies for this review are presented in Fig. 1.

### Assessment of the quality of the included articles

Checklists from the National Institute of Clinical Excellence (NICE) were utilized to assess the quality of the qualitative or quantitative articles that were included [4]. The checklists include frameworks for assessing the population, design, validity, bias, and reliability of a study. The Quality Appraisal Checklist for Quantitative Studies was used to assess the included quantitative studies and the quantitative portion of the mixed-methods studies (Additional file 1: Table S1). Most of the studies had a representative population and a sound study method, but in the data analysis section not many used multiple explanatory analysis. Since all were descriptive studies, none included follow-ups or comparison interventions.

The Qualitative Apprasial Checklist for Qualitative Studies was used to assess the included qualitative studies and the qualitative portion of the mixed-methods studies (Additional file 1: Table S2). Most of the studies had a sound study design and relevant findings. However, many did not include an explanation of the theoretical approach that was used to guide the study or details on how the data were anlayzed, thus raising the question of the whether the analysis was rigorous. On the basis of this assessment, the studies/reports were rated as being of moderate to good quality.

The opinion papers were not subjected to a quality evaluation. The opinion-based papers were all authored by influential healthcare professionals who were faculty members in schools of medicine, nursing, and midwifery, and all were retained in this review. The key components of each report were extracted and tabulated according to the names of the authors, the year of publication, the country/countries where the study was conducted, the design of the study, the target population, the sample size, and a summary of the main results (Additional file 2).

## Maintext

### Types of stakeholders included in the literature

The included literature showed that those stakeholders who had an interest in and expressed an opinion on the issue of women having an autonomous choice on whether to undergo a cesarean section included maternity care providers (MCPs), pregnant women, and the general public. Among the 55 reports included in this review, 18 research articles reported only the views of MCPs, and another 2 included the views of both MCP and pregnant women, and one included the views of both MCP and the public. A total of 14 focused on the views of pregnant women, and 4 on those of the general public. The remaining papers consisted of 16 opinion papers written by professionals.

### Maternity care providers

#### Characteristics of the literature on maternity care providers

Nine out of the 21 studies (15 quantitative and 6 qualitative studies) reported the views of obstetricians or obstetric trainees, and 2 studied the views of midwives. The reports were predominantly conducted in Western countries (*n* = 15), including the United Kingdom (*n* = 2), North America (*n* = 4), Australia (*n* = 2), Sweden (*n* = 3), Italy (*n* = 2), and Denmark (*n* = 1). One was conducted in eight different countries in Europe. Only 6 studies were conducted in Asia, namely China (*n* = 1), Turkey (*n* = 2), Jordan (*n* = 1), Iran (*n* = 1), and Africa (*n* = 1).

The 16 opinion-based articles were all written by obstetricians (one was co-authored with a nurse educator) from Western countries, predominantly the USA (*n* = 8), the UK (*n* = 5), Canada (*n* = 1), Australia (*n* = 1), and Sweden (*n* = 1).

The 15 quantitative studies included a total of 5461 obstetricians, 537 midwives, and 622 multidisciplinary health professionals (the professionals included obstetricicans, midwives, and anesthetists, but they were not separately identified in these studies). The largest sample, consisting of 1530 obstetricians, was from a study conducted in eight countries in Europe [5]. The six qualitative studies targeted obstetricians and midwives (*n* = 3), obstetricians (*n* = 1), and multidisciplinary health professionals including public health nurses, anesthetistis, and managers (*n* = 2), Sample sizes ranged from 8 to 46 participants, with the largest samples comprised of midwives (*n* = 54) and obstetricians (*n* = 53). These qualitative studies explored the views of health professionals on why women requested a CS, and how information was provided that did or did not support the decision made by the women.

#### Views of maternity care providers on cesarean sections per maternal request

In all of the included studies, the MCPs were asked if they supported the autonomous choice of a cesarean section per maternal request (CSMR) or would agree to perform a CS upon request. The studies revealed that MCPs generally supported the involvement of women in making decisions throughout their pregnancy.

Obstetricians were the most supportive of CSMRs, but this varied across countries. Among the registered fellows of the Maine branch of the American College of Obstetricians and Gynecologists [3], 84.5% indicated that they would perform a CSMR [6]. In Australia, 77.3% of the 1032 obstetricians and 81% of the 258 obstetric trainees would agree to a CSMR in uncomplicated pregnancies [7]). The lowest level of support for a CSMR was among obstetricians from Spain and Canada, where only 15 and 23% of obstetricians respectively would agree to perform a CSMR [5, 8]. In the US, 10.2% of obstetricians reported that they routinely perform CSMRs [9].

It is interesting to note that while 57.9% of obstetricians in Italy would support freedom of choice for women on the issue of MOB, not as many of them (40.8%) would agree to a CSMR [10]. The reverse situation was reported in Turkey, where not as many of the obstetricians agreed with freedom of choice for women on MOB (40.1%), but 53% would agree to perform a CSMR [11].

Gender differences were noted between male and female obstetricians in their support for CSMRs [8, 12]. In Canada, male obstetricians (34%) were more likely than female obstetricians (16%) to agree to perform a CSMR [8]. A similar difference was found in Italy, where 48.3% of male and 33.3% of female obstetricians would agree to perform a CS upon request [12]. Experience also seemed to have a bearing on whether obstetricians were willing to perform a CSMR. Three studies reported that trainee obstetricians and obstetricians who had been qualified for less than 10 years were more likely to agree to a CSMR [6, 7, 13].

A qualitative study conducted in Iran on the views of obstetricians towards CSMRs revealed that despite the government’s promotion of vaginal birth (VB), there are no policies in place to monitor the outcomes of deliveries among practitioners [14]. The obstetricians involved rationalized that if complications were to occur during a birth, they would be queried on why a CS had not been performed, and could subsequently become involved in a lawsuit. The obstetricians considered the CS to be a convenient scheduled procedure, one that was less likely to attract litigation, while also generating more income. As a result, when women requested a CS, the obstetricians would provide the woman with a description of the benefits and potential complications of undergoing a CS, and allow them to make the decision [14]. In Africa, 88.9% of obstetricians said that they would accommodate a woman’s request for a CSMR for reasons of patient autonomy [15].

Studies were conducted on the views of healthcare professionals on the right of pregnant woman to choose a cesarean section as the mode of birth. A study of female healthcare workers in Turkey, including nurses, doctors, and hospital employees, reported that nearly one-third (37.8%) of them agreed that women should be given the right to choose the MOB [16]. The differences between the views of the various female healthcare workers were not reported in the study. However, a smaller percentage of midwives were reported to support CSMRs, in that only 22–23% of midwives in Sweden would support a CSMR as an informed choice [17, 18]. The midwives stated that while they agreed that women should be allowed to choose the MOB, it is the duty of midwives to provide information to guide the women in the direction of choosing what midwives perceive to be the safest MOB [19–23].

#### Recommendations of maternity care providers to women

Maternity care providers were reported to have a tendency to recommend to women the MOB that is consistent with their own personal preference [11]. They also gave women descriptions of the birth process that were consistent with their own personal opinion [14]. A study conducted in China reported that female MCPs who had given birth by CS were more likely to advise women to choose a CS than those who had had a VB (86.9% vs. 55.6%) [24]. These MCPs reported that they were satisfied with the birth process that they had personally undergone, and would recommend the same to others.

Studies indicate that there are variations in the personal preferences of MCPs towards CS across different countries. Low rates of preference for CS among obstetricians were reported in the UK (10%), Jordan (7%), and Denmark (1%) [13, 25, 26]. About one third of obstetricicans in Canada support CSMR [27]. In China, 69.7% of the MCPs had had their own baby delivered by CS, 49% of those by CSMR [24]. Over 50% of obstetricians in Turkey were reported to have chosen a CSMR for themselves or their partner, predominantly on the basis of concerns regarding perineal trauma and associated adverse outcomes such as pelvic organ prolapse [11]. In Denmark, 37.6% of obstetricians considered it a woman’s right to have a cesarean section without any medical indications, but those who had themselves undergone a noninstrumental vaginal birth were less likely to agree with this right [26].

#### Non-research opinion articles

The non-research opinion articles (*n* = 16) on views and positions on the choice of MOB were written by senior and influential members of the obstetric community. In 12 articles (80%), a clear statement was made supporting a woman’s right to choose the MOB, indicating acceptance of CSMRs. However, nine of these articles also recommended that obstetricians need to exercise caution before agreeing to a CSMR. These authors took the position that although women should have the right to choose their MOB, many cannot do so as they are shouldering responsibility for themselves and the baby [28]. The obstetricians should make a professional judgment of the individual’s health and the clinical situation when deciding on whether to comply with the request [29, 30]. In the other three opinion articles, the obstetricians stated that scientific evidence is currently lacking on whether a VB or CS is the safest MOB, so there is not enough evidence to support whether women are making a “well” informed autonomous choice [31–33]. In summary, although the majority of obstetricians supported the concept of CSMR, they felt that a CSMR should only be performed after attempts have been made to establish that a CS is indeed a suitable choice for the individual.

The discussions in these opinion papers ranged from the issue of the autonomy of women at the individual level to the appropriate use of available resources at the system level. It was emphasized that comprehensive informed consent cannot realistically be achieved because the evidence is lacking to support either MOB as superior [34, 35]. The debate is not simply about the rights of women, but also about the demands on the health service system [36, 37]. As a result there are conflicting opinions on CSMRs, depending on whether obstetricians prioritize the free choice of the individual or highlight health service resource limitations in the debate on CSMRs.

Obstetric and/or midwifery organizations in many countries such as the USA, the UK, and even Hong Kong have issued position statements supporting the involvement of women in making decisions on pregnancy care [4, 38–40]. These organizations have clear recommendations and guidelines for obstetricians on CSMRs, including on the need to give a comprehensive explanation and counseling based on the most up-to-date evidence available for women who request a CS. Once counseling has been provided, if the CS request is maintained and informed consent has been obtained, it is considered reasonable to perform the CSMR [4, 38, 40, 41]. However, as there is no mechanism to monitor the implementation, whether practicing obstetricians are adhering to these recommendations is unknown.

### Views of pregnant women on cesarean sections per maternal request

There were 16 studies (nine quantitative, five qualitative, and two mixed-methods studies) examining the views of pregnant women on different modes of birth, with 14 focusing solely on pregnant women, and two on pregnant women and MCPs.

There were a total of 4672 women in the quantitative studies. A total of 35.9% of the women were from North America (1677), 26.1% (*n* = 1218) from Asian countries including China, Singapore, Thailand, and Turkey, 18.0% (*n* = 843) from West Africa, and 10.9% (*n* = 507) from the UK and Australia. The qualitative studies included interviews with a total of 336 women, most from Western countries.

#### A pregnant woman’s choice vs. an obstetrician’s responsibility

The majority of the studies reported that women wanted to have the freedom to choose their MOB. However whether women fully understand the question posed to them on “who should choose the MOB” is worth questioning.

In the quantitative studies, when women were asked a simple question on their right and freedom to make a decision on the MOB, the majority of pregnant women from Europe (88%), the USA (68–85.9%), and Singapore (71%) indicated that they should have the right to choose the MOB for themselves [42–45]. However, only one-third (32%) of pregnant women in Thailand indicated that they wished to choose the MOB [46]. A study in Canada found that 13% of nulliparas would choose CSMR if given the freedom to do so, while the figure was only 5% for multiparas [47]. Around 50–70% of pregnant women in the UK considered it the responsibility of obstetricians to decide whether a CS is necessary for the safety of the mother or baby [48].

Contradictory findings were noted within the same studies on whether the decision on a CS should be made autonomously by the pregnant woman or be the responsibility of her obstetrician. One study found that while 68% of women agreed that they should have the right to make the decision on a CS, 69% of them believed that women should follow the advice of obstetricians [44]. Another study revealed that 85.9% of women agreed that they should choose the MOB, but 79.6% of them also indicated that the decision on a CS was the obstetrician’s responsibility [45]. It is interesting to note that while 95% of women in US did not think that a CSMR is advisable, 75% of them believed that the decision should be up to the women themselves [49].

Indeed, the findings of the qualitative studies revealed that the majority of women believed that the decision on a CS should be made by obstetricians. Three qualitative studies and the qualitative section of a mixed-methods study conducted in Scotland, the UK, Australia, and Argentina [19, 22, 48, 50] reported that pregnant women sought to make the decision together with their obstetricians, rather than on their own. In a focus group interview study, women from Argentina (*n* = 29) actually considered obstetricians to be responsible for making decisions regarding a CS [51].

#### Is mode of birth the autonomous decision of women?

Whether women actually made an autonomous decision on MOB was explored in the studies. The studies were conducted in Australia, the UK, the USA, West Africa, and China.

Women described being autonomous in their decision on MOB [19, 52]. Women who perceived that they had made an autonomous decision indicated higher levels of satisfaction with their birth [52]. However, statements made by women about the process of making a decision on their MOB, showed that the women had followed the advice of their obstetrician [19]. These women said that, based on the advice of their obstetrician, they had chosen to undergo a CS because of the predictability and safety of the procedure for their baby and themselves, and that a VB involved unknown risks and unexpected outcomes. This indicated that even when women thought that they had made an autonomous decision, they had in fact followed the advice of their obstetrician. It was unclear whether the MOB was reflective of the women’s preference prior to the consultation with their obstetrician or was more of a reflection of the preference and opinion of their obstetrician.

A study conducted in Shanghai, China, reported that women who chose to have a CSMR were more likely than women who planned for a VB to have had the topic raised by an MCP who suggested that they undergo a CS [53]. Pregnant women who had indicated a preference for a VB but who had undergone a CS on the suggestion of their prenatal doctor/obstetrician, were 20 to 26 times more likely to change their mind and request a CSMR [53].

In making a decision about the MOB, women seemed to struggle over a decision that would have an impact on their unborn child and themselves [48]**.** In West Africa, more than 50% of women said that they would have wanted to ask for a CSMR, but were afraid that their request would be criticized, mainly by their own husbands [54]. Women tended to choose the MOB that was presented to them by MCP as being sensible and safe, placing the safety of their baby as the priority [19]. Women in Turkey considered VB to be a natural way of giving birth, and as one that helps to rid the body of waste products [55]. Explicit or implicit recommendations by their obstetrician, pointing out the risks to their baby of a particular MOB, were likely to result in the women following the advice of their obstetrician [48, 52].

### Public opinion

Five studies explored the attitudes of the general public towards the choice of MOB. The studies included women of childbearing age who were not pregnant (*n* = 3) and a survey of the general public (*n* = 2). The largest study was conducted in Sweden, where the participants (*n* = 1066) were asked about their views on decision-making relating to MOB [56]. The remaining studies were conducted in the USA, Canada, Australia, and Turkey, and comprised a total of 1160 participants.

Women among the general public who were not pregnant at the time of survey, were less supportive of autonomous choice than women who were pregnant. Only 38% of women from Sweden and 19% of women from Australia would agree that women should be allowed to choose a CS under any circumstances [57]. Young women were likely to be supportive of CSMRs and to believe that an increasing number of women of their generation would request a CSMR [58].

Between 28 and 44% of the general public agreed with CSMRs [16, 56, 58, 59]. The highest proportion of public support for CSMRs came from the USA, where 44% of those surveyed indicated that they were supportive of CSMRs, and agreed that insurance companies should cover the costs of CSMRs [59]. The lowest proportion of public support came from Canada, where 28.6% of women aged 18–24 indicated that they had a favorable attitude toward CSMRs, while 59% were concerned that CSMRs would impose a greater expense on the healthcare system [58].

## Conclusions

This review of the literature presented a debate of whether women has the choice and the freedom to choose a cesarean as the mode of birth for their baby from the views of various stakeholders.

It was found that the USA (84.5%) and Australia (77.3%) had the highest proportion of obstetricians who would perform a CSMR in uncomplicated pregnancies, while Spain and Canada had the lowest proportion (15 and 23%) [5–7]. A relatively smaller percentage of midwives (22–23%) in Sweden would support a CSMR as an informed choice [17, 18]. Maternity care professionals tended to recommend the MOB that was consistent with their own personal preference, and to provide descriptions of the birth process consistent with their own personal opinions [11, 26). It is clear that obstetricians are more supportive than midwives of the decision to carry out a cesarean section at the request of the pregnant woman.

The views of pregnant women on whether they should have the right to choose their MOB varied in different countries. While a large proportion of pregnant women (68–85.9%) in Europe and the USA believed that they should have the right to choose the MOB [11, 44, 45, 49], only one-third of those in Thailand agreed [46]. However, while 68–85.9% of pregnant women from the USA agreed that they should have the right to make the decision on a CS, 69–79.6% of them also believed it is the responsibility of their obstetrician to make the decision [44, 45]. In another study, also conducted in the US (New York), 95% of women did not consider CSMR to be advisable [49]. Pregnant women want to protect their baby and themselves, meaning that they will rely on the implicit or explicit recommendation of maternity care professionals. When the majority of pregnant women who perceived that they had an autonomous choice in the mode of birth were in fact influenced by their perception of maternity care professionals and by the information that these professionals provided.

The decision-making process of women relating to MOB may be more complicated than a simple discussion relating to freedom of choice for pregnant women, as it may also depend on their perception of the risks involved, how information was communicated by their MCP, and on the ability of the women to interpret the information that they had been given and to accept responsibility for their own decision. However, most of the included studies are largely descriptive, and do not examine the process by which the women made decisions throughout their pregnancy. For women, depending on their perception of the risks and on their ability to interpret the information communicated to them by maternity care professionals, the decision on the mode of birth is a more complex one than that of simple free choice.

In countries where there were well-developed private healthcare systems, higher percentages of stakeholders, particularly obstetricians, were supportive of CS per request [59]. In countries with public healthcare systems, smaller percentages of stakeholders were supportive of CSMR, out of concern that CSMR would impose a greater burden on the healthcare system [58]. One should note that maternity services in the two countries differ, with the USA having a well-developed private insurance system, whereas Canada has universally funded maternity care with patients paying for additional health services such as private rooms. This result indicated that the general public’s views towards a woman’s freedom to choose the MOB appeared to be related to the healthcare system, specifically to whether CSMRs are publically or privately funded.

Professional organizations have clear recommendations and guidelines for obstetricians on CSMR. In a publication of the Committee on Obstetric Practice of the ACOG [60] some outcome variables are outlined that favor planned vaginal birth and cesarean birth, with evidence of moderate quality regarding the different MOBs for a term singleton with a vertex presentation. It is further asserted that the motivation behind a CSMR should not be to avoid labor pain, nor should aCSMR be appropriate for women who desire several children. The report concluded by stating that in the absence of medical indications, a vaginal birth is safe and appropriate and should be recommended to patients. If obstetricians are approached by women for a CSMR, they are to provide a comprehensive explanation of the most up-to-date evidence available to women who request a CS. The request must also be followed by the woman’s signed consent; only then would it be considered reasonable to perform a CSMR [4, 38, 40, 41]. However, none of the studies investigated the adherence of obstetricians to the recommendations and guidelines, or the facilitators and barriers to the implementation of those recommendations and guidelines.

### Limitations

There are limitations to this review. Only articles published in English were included in this study. Useful information and study results published in other languages could therefore have been missed. In this review, only manuscripts of acceptable quality based on the appraisal quality checklist of the NICE were included, while unpublished studies, editorials, conference abstracts, and theses/dissertations on this and related topics were excluded, inevitably contributing to publication bias. The included studies, both quantative and qualitative, cover the views of various stakeholders towards the choice of a cesarean section as the mode of birth. The various methods adopted in these studies make it impossible to make more in-depth comparisons.

### Summary

The debate over whether women should have the right to decide on their MOB is not a simple one. Although an increasing number of women seem to be requesting a CS, in some of those cases their decision was explicitly or implicitly supported by their obstetrician.

This review of the literature sheds light on the implications for practice and future research. There is general support among women for choosing a CS and clear position statements and guidelines from professional bodies, but limited discussion on how the guidelines are implemented in practice. There is also no mechanism to monitor implementation; thus, whether practicing obstetricians are adhering to these recommendations is unknown. A standard protocol that promotes adherence to the recommendations and guidelines of professional organizations should be developed for use in maternity clinics. When information and counseling are provided to women who request a CS, there should be a record of the action taken and of the informed consent signed by the women. Women should be provided with update-to-date evidence to help them make an informed choice [31, 38, 61].

Research should be conducted in the future to explore the process by which pregnant women make the decision on MOB. The process of coming to a decision is not a simple or convenient one. While women may begin by taking into account the safety of their baby and themselves, they may also hear stories from relatives and friends, and actively search for information from various different sources to explore the pros and cons of different modes of birth. Women are also likely to have the topic raised by their obstetrician, which can influence their decision. A study has reported that women whose obstetrician suggested that they undergo a CS were more likely to change their mind and request a CSMR [53]. Longitutional studies should also explore how women process or interpret the information that is provided to them, and how this influences their decision-making process during the pregnancy.

## Additional file

Additional file 1: **Table S1.** NICE appraisal checklist for quantitative studies. **Table S2.** NICE appraisal checklist for qualitative studies. (DOCX 71 kb)

Additional file 2: Summary of studies on the views of stakeholders on the choice of mode of delivery. (DOCX 68 kb)

## Data Availability

Not applicable, the data of the literature included in this review are summarized in a table and submitted together with this manuscript

## Abbreviations

CS: Cesarean section
CSMR: Cesarean section per maternal request
MCP: Maternal care providers
MOB: Mode of mirth
NICE: National Institute of Clinical Excellence
UK: United Kingdom
USA: United States of America
VB: Vaginal birth

## References

[1] Goldberg H (2009). Informed decision making in maternity care. J Perinat Educ.

[2] Kaimal A, Kuppermann M (2012). Decision making for primary cesarean birth: the role patient and provider preferences. Semin Perinatol.

[3] American College of Obstetricians and Gynecologists. Surgery and Patient choice: the ethcis of decision making [ACOG Committee Opinion no 289] Obstet Gynecol 2003;102:1101–6.https://www.ncbi.nlm.nih.gov/pubmed/14672494. 10.1016/j.obstetgynecol.2003.09.030 14672494

[4] National Institute for Health and Care Excellence. Methods for the development of NICE public health guidance (third edition). 2012. retrieved from https://www.nice.org.uk/process/pmg4/chapter/appendix-h-quality-appraisal-checklist-qualitative-studies. Accessed 25 July 2019. 27905711

[5] Habiba M, Kaminiski M, Da Frè M, Marsal K, Bleker O, Librero J, Grandjean H, Gratia P, Guaschino S, Heyl W, Taylor D, Cuttini M (2006). Caesarean section on request: a comparison of obstetricians' attitudes in eight European countries. BJOG..

[6] Wax JR, Cartin A, Pinette MG, Blackstone J (2005). Patient choice cesarean--the Maine experience. Birth..

[7] Robson SJ, Tan WS, Adeyemi A, Dear KBG (2009). Estimating the rate of cesarean section by maternal request: anonymous survey of obstetricians in Australia. Birth..

[8] Farrell SA, Baskett TF, Farrell KD (2005). The choice of elective cesarean birth in obstetrics: a voluntary survey of Canadian health care professionals. Int Urogynecol J Pelvic Floor Dysfunct.

[9] Bettes BA, Coleman VH, Zinberg S, Spong CY, Portnoy B, DeVoto E, Schulkin J (2007). Cesarean birth on maternal request: obstetrician-gynecologists' knowledge, perception, and practice patterns. Obstet Gynecol.

[10] Mancuso A, De Vivo A, Fanara G (2006). Cesarean section on demand: are there differences related to obstetricians’ gender?. J Matern-Fetal Neonatal Med.

[11] Arikan DC, Özer A, Arikan I, Coskun A, Kiran H (2011). Turkish obstetricians' personal preference for mode of birth and attitude toward cesarean birth on maternal request. Arch Gynecol Obstet.

[12] Mancuso A, Settineri S, Fanara G, De Vivo A, Caruso C, Fattori A, Monaco I (2006). Confidential survey on cesarean section on request: obstetricians’ personal experience in Sicily. Acta Obstet Gynecol Scand.

[13] Lataifeh I, Zayed F, Al-Kuran O, Al-Mehaisen L, Khriesat W, Khader Y (2009). Jordanian obstetricians' personal preference regarding mode of birth. Acta Obstet Gynecol Scand.

[14] Bagheri A, Masoudi Alavi N, Abbaszadeh F (2013). Iranian obstetricians’ views about the factors that influence pregnant women's choice of birth method: a qualitative study. Women Birth..

[15] Obed JY, Bako BG, Agida TE, Nwobodo EI (2013). Caesarean birth on maternal request: consultants' view and practice in the west African sub region. J West Afr Coll Surg.

[16] Koken G, Cosar E, Sahin FK, Tolga Arioz D, Duman Z, Aral İ (2007). Attitudes towards mode of birth and cesarean on demand in Turkey. Int J Gynecol Obstet.

[17] Danerek M, Maršál K, Cuttini M, Lingman G, Nilstun T, Dykes A (2011). Attitudes of midwives in Sweden toward a woman's refusal of an emergency cesarean section or a cesarean section on request. Birth.

[18] Gunnervik C, Josefsson A, Sydsjö A, Sydsjö G (2010). Attitudes towards mode of birth among Swedish midwives. Midwifery..

[19] Bryant J, Porter M, Tracy SK, Sullivan EA (2007). Caesarean birth: consumption, safety, order, and good mothering. Soc Sci Med.

[20] Jacobson CH, Zlatnik MG, Kennedy HP, Lyndon A (2013). Nurses’ perspectives on the intersection of safety and informed decision making in maternity care. J Obstet Gynecol Neonatal Nurs.

[21] Karlström A, Engström-Olofsson R, Nystedt A, Thomas J, Hildingsson I (2009). Swedish caregivers’ attitudes towards caesarean section on maternal request. Women Birth..

[22] Mander R, Melender H (2009). Choice in maternity: rhetoric, reality and resistance. Midwifery..

[23] Kennedy HP, Grant J, Walton C, Sandall J (2013). Elective caesarean birth: a mixed method qualitative investigation. Midwifery..

[24] Ouyang YQ, Zhang Q (2013). A study on personal mode of birth among Chinese obstetrician-gynecologists, midwives and nurses. Arch Gynecol Obstet.

[25] Lightly K, Shaw E, Dailami N, Bisson D (2014). Personal birth preferences and actual mode of birth outcomes of obstetricians and gynaecologists in south West England; with comparison to regional and national birth statistics. Eur J Obstet Gynecol.

[26] Bergholt T, Ostberg B, Legarth J, Weber T (2004). Danish obstetricians' personal preference and general attitude to elective cesarean section on maternal request: a nation-wide postal survey. Acta Obstet Gynecol Scand.

[27] Lamb F, Pasquier J (2010). Cesarean birth by maternal request: surveys of obstetricians. Birth.

[28] Jomeen J (2012). The Pardox of choice in maternity care. J Neonatal Nurs.

[29] Goodin M, Griffiths M (2012). Caesarean section on demand. Obstet Gynaecol Reprod Med.

[30] Minkoff H (2006). The ethics of cesarean section by choice. Semin Perinatol.

[31] Capitulo K, Klein VR (2010). Should pregnant women be able to choose elective cesarean as a birth option?. Am J Matern Child Nurs.

[32] Kalish RB, McCullough LB, Chervenak FA (2008). Patient choice cesarean birth: ethical issues. Curr Opinion Obstet Gynecol.

[33] Watkins L, Weeks AD (2010). Providing information to pregnant women: how, what, and where?[editorial]. Obstet Anesth Dig.

[34] Shek KL, Dietz HP (2013). Elective caesarean birth - the right choice for whom?. Curr Women's Health Reviews.

[35] Leeman LM, Plante LA (2006). Patient-choice vaginal birth?. Ann Fam Med.

[36] Kapfhamer JD, Menon S, Spellecy R (2012). The importance of risk tolerance in maternal autonomy. Am J Bioeth.

[37] Klein MC (2012). Cesarean section on maternal request: a societal and professional failure and symptom of a much larger problem. Birth.

[38] Caughey Aaron B., Cahill Alison G., Guise Jeanne-Marie, Rouse Dwight J. (2014). Safe prevention of the primary cesarean delivery. American Journal of Obstetrics and Gynecology.

[39] Midwives Council of Hong Kong. Handbook for Midwives. 2014. http://www.mwchk.org.hk/docs/Handbook_for_Midwives_e.pdf. Accessed 25 July 2019.

[40] D'Souza R (2013). Caesarean section on maternal request for non-medical reasons: putting the UK national institute of health and clinical excellence guidelines in perspective. Best Pract Res Clin Obstet Gynaecol.

[41] Wiklund I, Andolf E, Lilja H, Hildingsson I (2012). Indications for cesarean section on maternal request – guidelines for counseling and treatment. Sex Reprod Healthcare.

[42] Chong ESY, Mongelli M (2003). Attitudes of Singapore women toward cesarean and vaginal deliveries. Int J Gynecol Obstet.

[43] Atan SU, Duran ET, Kavlak O, Donmez S, Sevil U (2013). Spontaneous vaginal birth or caesarean section? What do Turkish women think?. Int J Nurs Pract.

[44] Romero ST, Coulson CC, Galvin SL (2012). Cesarean birth on maternal request: a western North Carolina perspective. Matern Child Health J.

[45] Pevzner L, Preslicka C, Bush MC, Chan K (2011). Women's attitudes regarding mode of birth and cesarean birth on maternal request. J Matern Fetal Neonatal Med.

[46] Yamasmit W, Chaithongwongwatthana S (2012). Attitude and preference of Thai pregnant women towards mode of birth. J Med Assoc Thail.

[47] Pakenham Susan, Chamberlain Susan M., Smith Graeme N. (2006). Women’s Views on Elective Primary Caesarean Section. Journal of Obstetrics and Gynaecology Canada.

[48] Kingdon C, Neilson J, Singleton V, Gyte G, Hart A, Gabbay M, Lavender T (2009). Choice and birth method: mixed-method study of caesarean birth for maternal request. BJOG.

[49] Pevzner L, Goffman D, Freda MC, Dayal AK (2008). Patients' attitudes associated with cesarean birth on maternal request in an urban population. Am J Obstet Gynecol.

[50] Jenkins MG, Ford JB, Morris JM, Roberts CL (2014). Women's expectations and experiences of maternity care in NSW – what women highlight as most important. Women Birth.

[51] Liu NH, Mazzoni A, Zamberlin N, Colomar M, Chang OH, Arnaud L, Althabe F, Belizan JM (2013). Preferences for mode of birth in nulliparous Argentinean women: a qualitative study. Reprod Health.

[52] Wittmann-Price RA, Fliszar R, Bhattacharya A (2011). Elective cesarean births: are women making emancipated decisions?. Appl Nurs Res.

[53] Deng W, Klemetti R, Long Q, Wu Z, Duan C, Zhang W, Ronsmans C, Zhang Y, Hemminki E (2014). Cesarean section in Shanghai: Women's or healthcare provider's preferences?. BMC Pregnancy Childbirth.

[54] Okonkwo NS, Ojengbede OA, Morhason-Bello IO, Adedokun BO (2012). Maternal demand for cesarean section: perception and willingness to request by Nigerian antenatal clients. Int J Women's Health.

[55] Boz I, Teskereci G, Akman G (2016). How did you choose a mode of birth? Experiences of nulliparous women from Turkey. Women Birth..

[56] Hogberg U, Lynoe N, Wulff M (2008). Cesarean by choice? Empirical study of public attitudes. Obstet Anesth Dig.

[57] Haines H, Rubertsson C, Pallant JF, Hildingsson I (2010). Womens' attitudes and beliefs of childbirth and association with birth preference: aA comparison of a Swedish and an Australian sample in mid-pregnancy. Midwifery.

[58] Gallagher F, Bell L, Waddell G, Benoît A, Côté N (2012). Requesting cesareans without medical indications: an option being considered by young Canadian women. Birth..

[59] Thurman A, Zoller J, Swift S (2004). Non-pregnant patients’ preference for birth route. Int Urogynecol J.

[60] The American College of Obstetricians and Gynecologists. Committee on Obstetric Practice. Cesarean Birth on Maternal Request. 2013 (Reaffirmed 2017). No 559. Obstet Gynecol 2013;121:904–907. https://www.ncbi.nlm.nih.gov/pubmed/23635708. Accessed 25 JUly 2019.

[61] Duckworth S (2008). Should maternal choice be an indication for caesarean section?. Int J Surg.

