# Supplementary material for: Is it the decision of women to choose a cesarean section as the mode of birth? A review of literature on the views of stakeholders
Source: BMC Pregnancy Childbirth. 2019 Aug 9;19:286. doi: 10.1186/s12884-019-2440-2 (PMC6688235; doi:10.1186/s12884-019-2440-2)
Supplement: Supplementary file 1 — Table S1. NICE appraisal checklist for quantitative studies. Table S2. NICE appraisal checklist for qualitative studies. (DOCX 71 kb) [file 12884_2019_2440_MOESM1_ESM.docx]

**Additional file 1**

**Table S1: NICE Appraisal checklist of included quatitative studies**

| Study | **1. Population** | | | **2. Method of selection** | | | | | **3. Ooutcomes** | | | | | **4. Analysis** | | | | **5. Summary** | |
| --- | --- | --- | --- | --- | --- | --- | --- | --- | --- | --- | --- | --- | --- | --- | --- | --- | --- | --- | --- |
| Items*: | **1.1** | **1.2** | **1.3** | **2.1** | **2.2** | **2.3** | **2.4** | **2.5** | **3.1** | **3.2** | **3.3** | **3.4** | **3.5** | **4.1** | **4.2** | **4.3** | **4.4** | **5.1** | **5.2** |
| Arikan D, et al (2011) | ++ | ++ | + | + | + | + | ++ | ++ | ++ | ++ | ++ | NA | NA | NA | + | + | + | + | ++ |
| Bettes BA, et al. (2007) | ++ | ++ | + | ++ | ++ | ++ | ++ | ++ | ++ | ++ | ++ | NA | NA | NA | + | ++ | + | ++ | ++ |
| Bergholt T, et al. (2004) | ++ | ++ | ++ | ++ | ++ | ++ | ++ | ++ | ++ | ++ | ++ | NA | NA | NA | + | ++ | ++ | ++ | ++ |
| Danerek M, et al. (2011) | ++ | ++ | + | ++ | ++ | + | + | + | + | + | + | NA | NA | NA | + | + | + | + | + |
| Farrell S, et al. (2005) | ++ | + | + | + | + | + | + | + | ++ | + | + | NA | NA | NA | ++ | + | + | + | + |
| Gunnervik C, et al. (2010) | + | + | ++ | ++ | ++ | ++ | + | ++ | + | + | + | NA | NA | NA | + | - | - | ++ | + |
| Habiba M, et al. (2006) | + | + | + | + | + | + | + | ++ | + | + | + | NA | NA | NA | + | + | + | ++ | + |
| Koken G, et al. (2007) | + | - | - | - | + | + | + | + | + | + | + | NA | NA | NA | - | + | + | + | + |
| Lataifeh I, et al. (2009) | ++ | + | + | + | + | + | + | + | + | + | + | NA | NA | NA | + | + | - | + | + |
| Mancuso A, et al. (2006) | + | + | + | + | + | + | + | + | + | + | - | NA | NA | NA | - | + | + | + | + |
| Mancuso A, et al. (2006) | + | + | + | + | + | - | - | + | + | + | + | NA | NA | NA | + | + | + | + | + |
| Obed JY, et al.(2013) | + | + | + | + | + | + | + | + | + | + | + | NA | NA | NA | + | + | + | + | + |
| Ouyang YQ, et al. (2013) | + | + | + | - | + | + | + | + | + | + | + | NA | NA | NA | + | + | + | + | + |
| Robson SJ, et al. (2009) | ++ | ++ | ++ | ++ | ++ | ++ | ++ | ++ | ++ | ++ | + | NA | NA | NA | + | + | + | + | + |
| Wax JR, et al. (2005) | ++ | ++ | ++ | ++ | ++ | ++ | ++ | ++ | ++ | ++ | ++ | NA | NA | NA | + | + | + | + | + |
| Atan SU, et al. (2013) | ++ | + | + | + | + | + | + | ++ | + | ++ | + | NA | NA | NA | + | + | + | + | + |
| Chong ESY, et al. (2003) | + | + | + | + | + | + | + | + | + | + | + | NA | NA | NA | + | + | + | + | + |
| Deng W, et al. (2014) | ++ | ++ | ++ | ++ | ++ | ++ | ++ | ++ | ++ | ++ | ++ | ++ | ++ | NA | ++ | ++ | ++ | ++ | ++ |
| Kingdon C, et al. (2009) | ++ | ++ | ++ | ++ | ++ | ++ | ++ | ++ | ++ | ++ | ++ | NA | NA | NA | ++ | ++ | + | ++ | + |
| Okonkwo NS, et al. (2012) | ++ | ++ | ++ | ++ | ++ | ++ | ++ | ++ | ++ | ++ | ++ | NA | NA | NA | + | + | + | + | + |
| Pakenham S, et al. (2006) | + | + | + | + | + | + | + | + | + | + | + | NA | NA | NA | + | + | + | + | + |
| Pevzner L, et al. (2008) | + | + | + | + | + | + | + | + | + | + | + | NA | NA | NA | - | + | + | + | + |
| Pevzner L, et al. (2011) | + | + | + | + | + | + | + | + | + | + | + | NA | NA | NA | - | + | + | + | + |
| Romero ST, et al. (2012) | ++ | ++ | ++ | + | ++ | ++ | + | ++ | + | + | + | NA | NA | NA | - | + | + | + | + |
| Wittmann et al. (2011) | + | - | + | - | + | + | + | - | + | + | - | NA | NA | NA | + | + | + | + | + |
| Yamasmit W, et al. (2012) | ++ | ++ | + | + | + | + | + | + | + | + | + | NA | NA | NA | ++ | + | + | + | + |
| Gallagher F, et al. (2012) | ++ | ++ | ++ | ++ | ++ | ++ | ++ | ++ | ++ | ++ | ++ | NA | NA | NA | + | ++ | ++ | ++ | ++ |
| Haines H, et al. (2012) | ++ | ++ | ++ | ++ | ++ | ++ | ++ | ++ | ++ | ++ | ++ | NA | NA | NA | + | ++ | ++ | ++ | ++ |
| Hogberg U, et al. (2008) | **++** | **+** | ++ | **++** | **++** | **++** | **++** | **++** | **++** | **++** | **++** | **NA** | **NA** | **NA** | **++** | **++** | **++** | **++** | **++** |
| Thurman A, et al. (2004) | ++ | + | + | + | + | ++ | + | ++ | + | + | ++ | NA | NA | NA | + | + | + | + | + |

***Items:**

1.1 Is the source population or source area well described?

1.2 Is the eligible population or area representative of the source population or area?

1.3 Do the selected participants or areas represent the eligible population or area?

2.1 Selection of exposure (and comparison) group. How was selection bias minimised?

2.2 Was the selection of explanatory variables based on a sound theoretical basis?

2.3 Was the contamination acceptably low?

2.4 How well were likely confounding factors identified and controlled?

2.5 Is the setting applicable to the UK?

3.1 Were the outcome measures and procedures reliable?

3.2 Were the outcome measurements complete?

3.3 Were all the important outcomes assessed?

3.4 Was there a similar follow-up time in exposure and comparison groups?

**3**.5 Was follow-up time meaningful?

4.1 Was the study sufficiently powered to detect an intervention effect (if one exists)?

4.2 Were multiple explanatory variables considered in the analyses?

4.3 Were the analytical methods appropriate?

4.4 Was the precision of association given or calculable? Is association meaningful?

5.1 Are the study results internally valid (i.e. unbiased)?

5.2 Are the findings generalisable to the source population (i.e. externally valid)?

**Rating scales:** “++”conducted in such a way that minmise the risk of bias, “+”may not have addressed all potential sources of bias, “- “significant bias may persist. NR=not reported, NA=not applicable.

**Table S2: NICE appraisal checklist of qualitative studies**

| Study | Theorectical approach | | Study design and data collection | | Trustworthiness | | | Analysis | | | Findings | | Conclusion | Ethics | Overall assessment |
| --- | --- | --- | --- | --- | --- | --- | --- | --- | --- | --- | --- | --- | --- | --- | --- |
|  | 1. thorectical approach | 2. study purpose | 3. study design? | 4. data collection process | 5. researcher’s role clearly described? | 6. context clear? | 7. method reliable? | 8. rigorous in analysis | 9. rich data? | 10. anlaysis reliable? | 11. findings convenc-ing? | 12. relevant findings? | 13. adequate disucssion | 14. clear and coherent? | Quality |
| Evaluation outcomes | Appropriate  Inappropriate  Not sure | Clear  Unclear | Defensible  Indefensible | Defensible  Indefensible | Clearly described  Unclear  Not described | Clear  Unclear  Not sure | Reliable  Unreliable  Not sure | Rigorous  Not rigorous  Not sure/not reported | Rich  Poor  Not sure/not reported | Reliable  Unreliable  Not sure/not reported | Convincing  Not convincing  Not sure | Convincing  Not convincing  Not sure | Adequate  Inadequate  Not sure | Adequate  Inadequate  Not sure | **++** (mostly fulfilled)  **+** (some fulfilled)  - (few fulfilled, likley to alter conlcusion) |
| Bagheri A, et al. (2013) | Not sure | Clear | Defensible | Defensible | Not described | Clear | Reliable | Not reported | Rich | Not reported | Convencing | Convencing | Inadequate | Adequate | + |
| Bryant J, et al.(2007) | Not sure | Clear | Defensible | Indefensiable | Not described | Clear | Not sure | Not reported | Rich | Not reported | Convencing | Convencing | Inadequate | Adequate | + |
| Jacobson C, et al.(2013) | Appropriate | Clear | Defensible | Defensible | Clearly described | Clear | Reliable | Rigorous | Rich | Reliable | Convencing | Convencing | Adeqaute | Adequate | ++ |
| Karlström A, et al.(2009) | Appropriate | Clear | Defensible | Defensible | Clearly described | Clear | Reliable | Rigorous | Rich | Reliable | Convencing | Convencing | Adeqaute | Adequate | ++ |
| Kennedy HP, et al.(2013) | Appropriate | Clear | Defensible | Defensible | Clearly described | Clear | Reliable | Rigorous | Rich | Reliable | Convencing | Convencing | Inadeqaute | Adequate | ++ |
| Mander R, et al.(2009) | Appropriate | Clear | Defensible | Defensible | Clearly described | Clear | Reliable | Rigorous | Rich | Reliable | Convencing | Convencing | Adeqaute | Adequate | ++ |
| Boz I, et al.. (2016) | Appropriate | Clear | Defensible | Defensible | Clearly described | Clear | Reliable | Rigorous | Rich | Reliable | Convencing | Convencing | Adeqaute | Adequate | ++ |
| Jenkins MG, et al.(2014) | Appropriate | Clear | Defensible | Defensible | Not described | Clear | Reliable | Rigorous | Rich | Reliable | Convencing | Convencing | Adeqaute | Adequate | ++ |
| Kingdon C, et al.(2009) | Inappropriate | Clear | Defensible | Indefensible | Not clearly described | Clear | Unreliable | Not rigorous | Poor | unreliable | Not vonvencing | Convencing | Adeqaute | Adequate | + |
| Liu NH, et al.. (2013) | Not sure | clear | Defensible | Indenfensible | Not clearly described | clear | Reliable | Not rigorurs | Rich | Unreliable | Convencing | convencing | Adeqaute | Adequate | + |
| Wittmann- et al.(2011) | Not sure | clear | indefensible | indefensible | Not described | unclear | unreliable | Not rigorous | poor | unrelaible | Not convencing | Not convencing | inadeaute | inadequate | - |
