# Supplementary material for: Is it the decision of women to choose a cesarean section as the mode of birth? A review of literature on the views of stakeholders
Source: BMC Pregnancy Childbirth. 2019 Aug 9;19:286. doi: 10.1186/s12884-019-2440-2 (PMC6688235; doi:10.1186/s12884-019-2440-2)
Supplement: Supplementary file 2 — Summary of studies on the views of stakeholders on the choice of mode of delivery. (DOCX 68 kb) [file 12884_2019_2440_MOESM2_ESM.docx]

**Additional file 2 Summary of studies on the views of stakeholders on the choice of mode of delivery**

| Quantitative or mixed-method studies | | | | | | |
| --- | --- | --- | --- | --- | --- | --- |
| Study | **Country** | **Study design** | **Target population** | | | **Summary of the Main Results** |
|  |  |  | Obstetricians & Gynaecologists (O&G)/trainees or Midwives (MW) | Pregnant women | General public |  |
| Arikan D, Ozer A, Arikan I, Coskun A, Kiran H.  (2011) | Turkey | Quantitative, descriptive study | 387 O&G at a conference | - | - | 61.8% of obst. or their partner had CS.  88.7% of those had a CSMR.  Common reasons: perineal trauma.  40.8% believe that a woman has the right to choose a CSMR.  53% said that they would perform a CSMR.  Obst. < 44 yrs are more likely to choose a CSMR. |
| Bagheri A, Alavi N, Abbaszadeh F. (2013) | Iran | Qualitative, semi-structured interviews, inductive qualitative content analysis | 18 O&G and resident medical staff in 3 Kashan city hospitals | - | - | Six themes:  Relating to women – labour is painful, women do not consider a CS to be major surgery.  Relating to Obs. – labour is long, can manage the workload, more income, safer.  Relating to the healthcare system: don’t support in legal cases, no incentive for low CS rate.  Relating to culture and society: CS is modern, for rich people.  Complications of VB and CS: CSs are now safer than ever and more predictable than VB. |
| Bettes BA, Coleman VH, Zinberg S, Spong CY, Portnoy B, DeVoto E, Schulkin J. (2007) | USA | Quantitative mailes questionnaires | 1031 fellows of ACOG | - | - | -54% believe women have the right to cesarean delivery on maternal request  -53% have done CSMR  10.2% doing CSMR rountinely  Demale O& G endorsed more riskd to CSMR than male O&G |
| Bergholt T, Østberg B, Legarth J, Weber T. (2004) | Danish | Quantitative postal nation-wide survey | 155 O & G of the Danish Scoiety of O& G | - | - | -37.6% agreed with a woman's right to have an elective cesarean section on maternal request without any medical indication.  -O&G who had experienced a noninstrumental vaginal delivery themselves were less likely to agree with the woman's right to CSMR. |
| Bryant J, Porter M, Tracy SK, Sullivan EA. (2007) | Australia | Qualitative, in-depth interviews | 6 O&G  12 MW | 18 Pregnant women | - | Viewed informed consent as involving either guiding or giving information and then allowing the women to choose.  If the women did not listen to medical advice, when complications developed they were considered “bad mothers”.  CS can easily be presented as the safe and controlled option.  Midwives and O&G had differing concepts of safety and a good birth.  Women viewed themselves as autonomous and able to make decisions. |
| Danerek M. et al. (2011) | Sweden | Quantitative | 259 MW working in a delivery suite | - | - | 77% believed that if CSMR is for “her own choice” the O&G should refuse.  71-80% believed that if the CSMR is for previous trauma, the O&G should agree, but should try to  persuade the women to agree with the O&G’s recommendations. |
| Farrell S, Basket T, Farrell K. (2005) | Canada | Quantitative, questionnaire | 162 O&G at a conference | - | - | 23% approved of CSMR, with informed consent, for a nulliparous woman.  34% of men vs 16% of women.  55% of men vs 38% of women would describe CS as being protective of the pelvic floor. |
| Gunnervik C, Josefsson A, Sydsjo A, Sydsjo G. (2010) | Sweden | Quantitative, exploratory descriptive study | 278 MW in 2 counties | - | - | Midwives considered 11.5% about right for the CS rate.  Midwives < 50yrs were more likely to consider the rate too high.  32% of midwives working in AN clinics agreed that women should be able to request a CSMR compared to 14.7% of DS midwives. |
| Habiba M, Kaminiski M, Da Frè M, Marsal K, Bleker O, Librero J, Grandjean H, Gratia P, Guaschino S, Heyl W, Taylor D, Cuttini M. (2006) | European countries: Luxembourg, Netherland, Sweden, France, Germany, Italy, Spain, UK | Quantitative cross sectional survey | 1530 obstetricicans |  |  | -Agreed to CSMR was lowest in Spain (15%), France (19%) and Netherlands (22%); highest in Germany (75%) and UK (79%)  -fear of litigation was associated with physicians’ likelihood to agree to CSMR  -female O&Gs with children were less likely to agree to CSMR |
| Jacobson C, Zlatnik M, Kennedy H, Lyndon A. (2013) | USA | Qualitative, constructivist grounded theory | 46 Nurses and O&G | - | - | Informed consent was regarded by nurses as involving preventing harm, managing the flow of communication to what they believed was the right course of action.  O&G & some nurses viewed safety as a healthy mum & baby.  More nurses viewed safety as more important than autonomy . |
| Karlstrom A. et al. (2009) | Sweden | Qualitative, descriptive study | 25MWs and Obs | - | - | Themes were about respecting the woman’s choice and trying to avoid agreeing to CSMR. Where there was a previous traumatic birth or CS, CSMR was more readily accepted. Where there was fear of birth, CSMR was not seen as the best approach. They found it difficult to manage requests for CSMR. |
| Kennedy HP, Grant J, Walton C, Sandall J. (2013) | UK | Interpretive, qualitative | 34 Clinicians (obs, M/W, anaesthetists | - | - | Four themes:  1. The culture of CS  2. CS counselling  3. Perceptions of choice  4. Negotiating the rules |
| Koken G. et al. (2007) | Turkey | Quantitative, self-administered survey | 329 Female HCP | - | 347 General public | HCP outcomes: 48.1% preferred VB, 45.3% CSMR, and 37.8% believed that a woman should be able to request CSMR.  Public outcomes: 69.6% preferred VB, 20.6% CSMR, and 36.2% believed that a woman should be able to request a CSMR. |
| Lataifeh I. et al. (2009) | Jordan | Quantitative, self-administered questionnaire | 315 O&G | - | - | 7% preferred CS.  14.1% of O&G < 45 yrs supported CSMR.  3.7% of O&G > 55yrs supported CSMR. |
| Mancuso A. et al. (2006) | Italy | Quantitative, self-administered questionnaire | 271 O&G | - | - | 77.9% of respondents were male.  57.6% supported women choosing the MOD.  91.2% noted women with gestational anxiety.  79.8% noted increased anxiety in later pregnancy.  31.6% noted difficulty managing gestational anxiety. |
| Mancuso A, Vivo A, Fanara G. (2006) | Italy | Quantitative, self-administered questionnaire | 120  60 females and 60 males O&G | - | - | 79.9% stated fear of birth as motivation for CSMR.  96.6% vs 46.6% of male and female O&G respectively reported fear of childbirth in their patients. |
| Mander R, Melender H. (2009) | Scotland | Qualitative, hermeneutic, phenomenological, interviews | 4 Midwives, 2 public health nurses, and 2 managers in Finland and New Zealand | 4 Mothers | - | Background theme: trusting the system. Perceived women as wanting the safety of the system. Felt uncomfortable going against the system. |
| Obed JY, Bako BG, Agida TE, Nwobodo EI. (2013) | West Africa | Quantitative s  Self-adminsitered questionnaire | 90 O& G |  |  | - 81.2% have operated on at least a patient for CDMR  -88.9% opined that it is important to accommodate the feelings of the women and offer CDMR for the respect of the patient's autonomy. |
| Ouyang Y, Zhang Q. (2013) | China | Quantitative | Females working in O&G | - | - | Chose CSMR because it is safer, faster, more predictable, and don’t trust skills of colleagues in managing VB. |
| Robson S, Tan W, Adeyemi A, Dear K. (2009) | Australia | Quantitative, self-administered questionnaire | 1032 O&G  258 trainees | - | - | 3% CSMR in Australia.  77.3% would agree to CSMR.  If working in a public hospital, 87.5% would not discuss CSMR.  64.5% of trainees said that they would agree to CSMR once qualified; rates increased to 77% in the final year.  O&G < 10 yrs qualified were more likely to agree. |
| Wax J, Cartin A, Pinette M, Blackstone J. (2005) | USA | Quantitative, self-administered questionnaire | 110 O&G fellows of ACOG. | - | - | 84.5% would conduct CSMR.  21.1% preferred CS for self or partner.  42.9% of O&G < 35 interpreted the evidence as supporting CS in all cases vs 4.2% of older O&G.  Women < 35 were more likely to choose CSMR for themselves. |
| Atan S. et al. (2013) | Turkey | Quantitative, self-administered questionnaire | - | 342 Women 24 hrs postnatal | - | 88% believed that women should choose the mode of delivery. |
| Boz I, Teskereci T, Akman G. (2016) | Turkey | Phenomenological study |  | 29 nulliparous women |  | Vaginal births were considered under  the theme “natural but hard way” and caesarean sections under the theme “easy choice”. |
| Chong ESY, Mongelli M. (2003) | Singapore | Quantitative, questionnaire | - | 160 Women 28-40 weeks pregnant at a large teaching hospital | - | 3.7% preferred CS.  71% believed that women have the right to choose, but are  generally poorly informed about the benefits and risks of CSMR. |
| Deng W. et al. (2014) | China | Quantitative, self-administered questionnaire | - | 272 Postnatal women | - | 57% CS rate.  17% with medical indications, 40% with no medical indications.  Women who had CS remember it being suggested by their obstetrician. |
| Jenkins M, Ford J, Morris J, Roberts C. (2014) | Australia | Qualitative, semi-structured interviews, descriptive analysis, purposive sampling | - | 53 Pregnant women accessing a broad range of services, remote to tertiary | - | Women wanted care focused on them and to be informed.  They also wanted to be involved in the decision-making process. |
| Kingdon C. et al. (2009) | UK | Mixed-method, longitudinal cohort study, in-depth interviews | **-** | 454 Nulliparous pregnant women  153 for interviews | - | Women did not have autonomous choice and did not want it, felt that their baby’s health should come first, and thought of CSMR as being only for the rich or for those who had a previous traumatic birth.  55% believed that women should not have the right to choose VB under any circumstances, while 50% of women agreed or strongly agreed that the doctor should always decide if a CS is required.  Were happy not to have autonomous choice, as they would blame themselves if something went wrong. |
| Liu NH, Mazzoni A, Zamberlin N, Colomar M, Chang OH, Arnaud L, Althabe F, Belizan JM. (2013) | Argentina | Qualitative study |  | 29 Nulliparous pregnant women |  | Most preferred vaginal delivery (VD) due to cultural, personal, and social factors.  Women viewed CS as a medical decision of medical professioanls and often deferred decisions in the presence of medical indications. |
| Okonkwo NS, Ojengbede OA, Morhason-Bello IO, Adedokun BO. (2012) | West Africa | Quantitative, cross-sectional questionnaire |  | 843 antenatal clients |  | More than 50% of those willing to request CSMR would likely be critized, maily by their hsubands |
| Pakenham S, Chamberlain SM, Smith GN. (2006) | Canada | Quantitative,  questionnaire |  | 107 nulliparous women and 103 multiparous women |  | -Thirteen percent of nulliparas stated that they would choose CSMR if given the option, compared with 5% of multiparas  -perceived risks of vaginal delivery were commonly cited by both nulliparas and multiparas as reasons for requesting CSMR  - he risks of Caesarean section for the baby or for future pregnancies were the most commonly cited reasons to decline CSMRin both groups. |
| Pevzner L, Goffman D, Freda MC, Dayal AK. (2008) | USA | Quantitative,  questionnaire |  | 188 pregnant women in mid-trimester |  | -95% did not believe that CDMR was advisable  -1% of this group felt that the decision should be left up to the individual.  - 75% believed that the decision should be left up to the individual. |
| Pevzner L. et al. (2011) | USA | Quantitative, cross-sectional, self-administered questionnaire | - | 833 Women attending 2 hospitals for mid pregnancy ultrasound | - | 6.1% believed that CSMR is a good idea.  85.9% believed that women should be able to choose the MOD.  79.6% believed that the choice of CSMR should be available to everyone. |
| Romero S, Coulson, C., & Galvin, S. (2012) | USA | Quantitative, self-administered questionnaire | - | 396 Pregnant women | - | 68%: CSMR with informed choice.  69%: patients should follow their doctor’s advice.  25%: patients should pay for CSMR. |
| Wittmann-Price R, A, Fliszar R, Bhattacharya A. (2011) | USA | Mixed-method | - | 50 Pregnant women | - | Women perceived that they have a choice; the perception of choice increases satisfaction. |
| Yamasmit W. (2012) | Thailand | Quantitative, self-administered questionnaire | - | 415 Pregnant women | - | 32% believed they should be able to request a CSMR.  77.3% preferred VB.  The sample included women with previous CS. |
| Gallagher F, Bell L, Waddell G, Benoit A, Cote N. (2012) | Canada | Quantitative, self-administered questionnaire | - | - | 140 Nulliparous, non-pregnant women 18-24 yrs old at vocational school or university | 63% have heard of CSMR.  28.6% had a favourable attitude towards CSMR.  59% perceive VB as less expensive for the healthcare service.  59% believe that more women of our generation will want one. |
| Haines H, Rubertsson C, Pallant J, Hildingsson I. (2012) | Sweden / Australia | Quantitative, self-administered questionnaire | - | - | Women living in rural districts in Sweden (386) and Australia (123) | Swedish women held stronger views on birth and the right to determine birth.  Women should be able to have a CS under any circumstance (38.3 vs 19%); doctors should decide on CS (64.9% vs 47.1%). |
| Hogberg U, Lynoe N, Wulff M. (2008) | Sweden | Quantitative, self-administered questionnaire | - | - | 1066 Swedish public | 59%: women should be able to choose the MOD.  39%: women should be able to choose the MOD irrelevant of the reason.  32%: women should be able to decide on the MOD without being pressured.  68%: The doctor should decide when a CS required. |
| Thurman A, Zoller J, Swift S. (2004) | USA | Quantitative, self-administered questionnaire | - | - | 164 Women presented to gynaecological clinic for routine non-pregnancy care | 44% of women agreed with CSMR.  44% believed insurance companies should pay for CSMR. |

**Opinion-based Articles**

| Author | Country | Authors’ designations | Main statements |
| --- | --- | --- | --- |
| Capitulo K. (2010) | USA | O&G and Nurse Educator | Debated points for and against CSMR.  Women should be allowed to choose; present the evidence, and then it is their choice.  The evidence base does not support CSMR as being safer; the evidence list is so long that informed consent probably does not happen. |
| Caughey AB, Cahill AG , Guise J, Rouse D J. (2014) | USA | Expert committee | Policy statement from ACOG.  CSMR needs a full informed consent process.  The evidence base is not strong.  Women who request CSMR should receive counselling prior to agreeing with their request. |
| Duckworth S. (2008) | UK | O&G | The NHS cannot afford to provide all women with the choice to choose CSMR when there are no complications with their pregnancy and no solid evidence to support CS being superior to VB. |
| D'Souza R. (2013) | UK | O&G faculty | Education on the NICE guidelines for CSMR.  Discuss the reason for requesting a CSMR.  Document the discussion well.  Provide counselling and education to address issues.  If there is a disagreement, refer to a colleague. |
| Goodin M, Griffiths M. (2012) | UK | O&G trainee  Obstetricians | CSMR increasing.  Women request them due to fear and a previous traumatic delivery.  The evidence base is confusing: Women have the right to choose and O&G have the right to refuse.  O&G should ensure that women are well informed, listen to why the women are requesting a CSMR, and see that the women receive counselling if appropriate.  If, following this process, CSMR is the deemed to be the best mode of delivery for that particular woman, then it is appropriate to proceed. |
| Jomeen J (2012) | UK | O&G faculty | Women want to exercise choice, but shouldering dual responsibility for themselves and the baby means that they often do not.  HCPs need to see how they can make it a reality. |
| Kalish R, McCullough L, Chervenak F. (2008) | USA | O&G faculty | How should O&G respond to a CSMR request?  Is it good clinical practice to perform a CSMR?  Should CSMR be routinely offered to all pregnant women? |
| Kapfhamer J, Menon S, Spellecy R. (2012) | USA | O&G faculty | Maternal autonomy is not all that needs to be considered.  Obstetrics is unpredictable.  Risk means different things to different people. |
| Klein M. (2012) | USA | O&G faculty | HCPs are choosing CSMR to avoid genitourinary problems.  Training that focuses on the abnormal amplifies these concerns.  CSMR is based on fear and a poor evidence base. |
| Lamb F. (2010) | Canada | O&G | 30% of O&G had a favourable attitude towards CSMR.  15% trainees had a favourable attitude towards CSMR. |
| Leeman L, Plante L. (2006) | USA | O&G faculty | CSMR is increasing.  ACOG supports a woman’s right to choose.  Evidence is lacking to support a CSMR over a planned VB; CSMR may become common before we understand the risks.  Women who want breech VB or NBAC cannot get these choices. |
| Minkoff H. (2006) | USA | O&G faculty | O&G have an obligation to the mother and fetus.  O&G have a right to refuse requests.  Women have the right to request.  O&G should refuse or acquiesce based on their perception of the available data. |
| Schonfeld T. (2013) | USA | O&G | Educational article, CSMR not a simple discussion.  Need to consider safety and resource use, economics, clinical outcomes, social outcomes, and patients’ autonomy and values. |
| Shek K, Kietz H. (2013) | Australia | O&G faculty | There is not enough data to fully understand the benefits / risks of CSMR.  Women should be fully involved in the decision-making process. |
| Watkins L, Weeks A. (2010) | UK | Obstetricians | Women should be involved and want information to reduce anxiety.  Lack of involvement leads to less satisfaction. |
| Wiklund I, Andolf E, Lilja H, Hildingsson I. (2012) | Sweden | O&G | Literature review of the beneficial aspects in the response to CSMR.  If, following counselling, the woman continues to request a CSMR, it is reasonable to comply with the request. |

Abbreviations:

ACOG – American College of Obstetricians and Gynecologists

AN – Antenatal

CS – Caesarean Section

CSMR – Caesaerean Section on Maternal Request

DS – Delivery Suite

HCP – Healthcare provider

MW – Midwife

NBAC – Next Birth After Caesarean

NICE – National Institute of Clinical Excellence

O&G – Obstetricians and Gynaecologists

Obs – Obstetricians

RANZCOG – Royal Australian and New Zealand College of Obstetricians and Gynaecologists

VB – Vaginal birth
